# Adult conspecific density affects Janzen-Connell patterns by modulating the recruitment exclusion zones

**DOI:** 10.3389/fpls.2023.1079975

**Published:** 2023-06-27

**Authors:** Giuliano Bonanomi, Aleksandr Bobrovskikh, Fabrizio Cartenì, Stefano Mazzoleni, Francesco Giannino

**Affiliations:** ^1^ Department of Agricultural Sciences, University of Naples Federico II, Naples, Italy; ^2^ Task Force of Microbiome Studies, University of Naples Federico II, Naples, Italy; ^3^ Laboratory of Plant Growth Biomechanics, Institute of Cytology and Genetics Siberian Branch of Russian Academy of Sciences (SB RAS), Novosibirsk, Russia; ^4^ Task Force of Computational and Quantitative Biology, University of Naples Federico II, Naples, Italy

**Keywords:** plant population and community dynamics, plant-soil negative feedback, autotoxicity, species coexistence, forest diversity, individual-based modelling, numerical simulations

## Abstract

Plant-soil negative feedback (NF) is a well-established phenomenon that, by preventing the dominance of a single species, allows species coexistence and promotes the maintenance of biodiversity. At community scale, localized NF may cause the formation of exclusion zones under adult conspecifics leading to Janzen-Connell (JC) distribution. In this study, we explore the connection between adult density, either conspecifics or heterospecifics, on the probability of occurrence of JC distributions. Using an individual-based modelling approach, we simulated the formation of exclusion zones due to the build-up of NF in proximity of conspecific adult plants and assessed the frequency of JC distribution in relation to conspecifics and heterospecifics density ranging from isolated trees to closed forest stands. We found that JC recruitment distribution is very common in the case of an isolated tree when NF was strong and capable to form an exclusion zone under the parent tree. At very low NF intensity, a prevalence of the decreasing pattern was observed because, under such conditions, the inhibitory effect due to the presence of the mother tree was unable to overcome the clustering effect of the seed dispersal kernel. However, if NF is strong the JC frequency suddenly decreases in stands with a continuous conspecific cover likely as a result of progressive expansion of the exclusion zone surrounding all trees in closed forest stands. Finally, our simulations showed that JC distribution should not be frequent in the case of rare species immersed in a matrix of heterospecific adults. Overall, the model shows that a plant suffering from strong NF in monospecific stands can rarely exhibit a recruitment pattern fitting the JC model. Such counterintuitive results would provide the means to reconcile the well-established NF framework with part the forest ecologists’ community that is still skeptical towards the JC model.

**Synthesis:**

Our model highlights the complex interconnection between NF intensity, stand density, and recruitment patterns explaining where and why the JC distribution occurs. Moreover, predicting the occurrence of JC in relation to stand density we clarify the relevance of this ecological phenomenon for future integration in plant community frameworks.

## 1 Introduction

More than 50 years ago two ecologists, Janzen (1970) and Connell (1971) in Central America and in Australia respectively, independently proposed the hypothesis that seeds and seedlings suffered a distance- and density-dependent mortality. Empirical observations reported that seed-fall obviously concentrated under the parent fruiting trees, whereas seedlings and saplings recruitment did not match the seed-fall kernel, unexpectedly peaking at a certain distance from the source tree. Their descriptive model consisted of two curves: the first showing seeds dispersal around a mother tree and the second reporting the survival probability of the seedlings as a function of distance from the same tree. Accordingly, a species was found unable to recruit under adult conspecifics because of the formation of an “exclusion area” where the mortality was disproportionately higher and not matching with the large seed availability (Janzen, 1970). Such phenomenon, preventing the dominance of a single species, was also recognized to allow species coexistence and to promote the maintenance of biodiversity in the ecosystems (Levi et al., 2019). In other words, field observations showed that a mortality agent operated in a distance, density-dependent, and species-specific way, impairing only the recruitment of the focal tree, with no effects on seeds and seedlings of other species.

Early reports of the Janzen-Connell (JC) recruitment distribution suggested that insects were killing all seeds falling under the canopy of the studied trees (Janzen, 1971). However, most later studies assessed only the occurrence of some indirect insect damages, missing the identification of a particular causative pest species (Basset et al., 2019). Other studies blamed the activity of vertebrates and mammals, but in most cases the activity of such animals cannot be related to the distance-dependent effects, considering their mobility over the forest floor (Song et al., 2021). In any case, predation is still considered as the main causal mechanism of the JC effect also in theoretical studies (Smith, 2022).

The role of soilborne and airborne pathogens has been also considered based on the assumption that pathogens propagules, including spores and sclerotia, may accumulate under the focal tree. For instance, Packer and Clay (2000) reported that the oomycete *Pythium* spp. disproportionately killed the seedlings rooted under the canopy of conspecific trees. Then, plant pathogens have been considered as main key factors in maintaining coexistence by causing local plant-soil Negative Feedback (NF), especially in wet ecosystems where oomycetes and fungi thrive (Agrios, 2005). However, most of the available experimental and field studies only reported pathogens damage, e.g., seeds covered by mold, seedlings wilting, leaf discoloration and spots, often missing the identification of the causal pathogenic agent and always without any assessment of consistency between the spatial distribution of the NF occurrence and the pathogens dispersal behavior.

Less popular is the hypothesis of the “exclusion zone” being generated by the accumulation of autotoxic chemical compounds. The study by Webb et al. (1967) was one of the first reporting that some unidentified autotoxic compound, killing seedlings of *Grevillea robusta*, could be the cause of the JC patterns reported in Australian forests. Autotoxicity has been reported for hundreds of plant species, mostly crops, and associated with the release of phytotoxic compounds during the decay of leaf and root litter, including a range of different compounds such as aromatic phenols, saponins, coumarins, and organic acids, among others (Singh et al., 1999). However, the autotoxicity hypothesis has suffered from a relevant objective weakness because all the above-mentioned compounds produce a generic phytotoxicity, thus unable to explain the species-specificity of the JC distributions. Moreover, no studies were able to identify and quantify the accumulation of any specific toxin under and around the focal tree. Despite these major problems, the autotoxicity hypothesis has continued to be inconsistently associated to generic allelopathic phytotoxic effects (Inderjit et al., 2021). Differently, autotoxicity found a logical explanation in the discovery that accumulation of fragmented extracellular DNA from decomposing leaf litter did cause extensive seed germination impairment and root damages to a broad range of higher plants, with toxic effects specifically limited to conspecifics (Mazzoleni et al., 2015). The evidence of such inhibitory effect by extracellular self-DNA was then recently confirmed and further investigated by means of whole-plant transcriptome and metabolomic profiling on the model plant *Arabidopsis thaliana* (Chiusano et al., 2021; Lanzotti et al., 2022).

**Figure 1:**
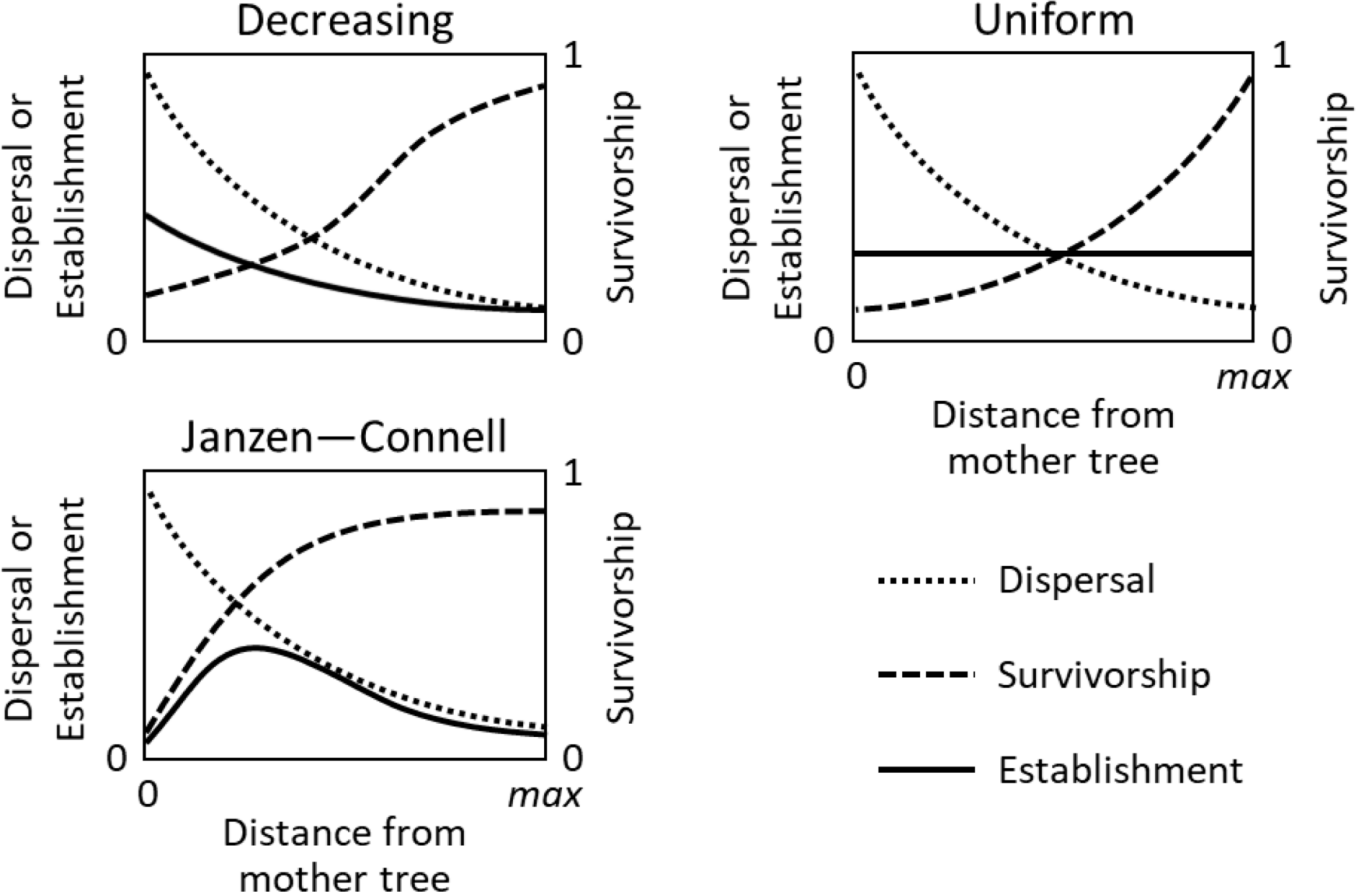
Common types of recruitment patters (adapted from Nathan and Casagrandi, 2004). Each plot shows the seed dispersal (dotted lines), survivorship (dashed lines) and establishment (solid lines) curves for the three most common recruitment patterns: monotonically decreasing (or Hubbell); uniform (or exact compensation); Janzen-Connell.

Irrespectively of the underlying mechanisms, several studies explored the consequences of the JC hypothesis using simulation models. Hubbell (1980) argued that disproportionately high seed densities under the parent tree would overcome the lower survival, thus resulting in monotonic recruitment patterns independent of distance from the focal plant. Later, Nathan and Casagrandi (2004) made a first systematic modelling exploration of the JC hypothesis. By using a mathematical model of distance and density-dependent seed mortality, the authors demonstrated that the net balance between seed dispersal and recruitment survival could generate all observed recruitment patterns, including both the hump-shaped typical JC distribution and monotonically decreasing (Hubbell) patterns (**Figure 1**). Later, Vincenot et al. (2017) presented an individual based model, at the scale of one focal plant, reporting that strong NF under a conspecific tree may overtake the seed dispersal kernel, thus creating an “exclusion area”. Moreover, the study also demonstrated that NF could produce an outward shift of the recruitment peak from seedlings to saplings, during a longer assessment of the recruitment process. More recently, Levi et al. (2019) changed the perspective from a single focal plant to ecosystem scale and, using high-performance computing and analytical models, demonstrated that distance-responsive natural enemies can maintain tropical forest diversity nearly indefinitely by favouring rare species. Moreover, the effect of NF at ecosystem level has been modelled, clearly explaining species coexistence (Bonanomi et al., 2005), its consistent relationship with biodiversity levels in different ecosystems in association with the rates of litter decomposition producing autotoxicity (Mazzoleni et al., 2010) and the formation of vegetation spatial patterns (Iuorio and Veerman, 2021)

Besides these robust modelling studies, the importance of the JC recruitment pattern is largely supported by many publications of empirical data from a broad range of ecosystems, including tropical (Mangan et al., 2010; Comita et al., 2014) and temperate forests (Fox, 1977; Packer and Clay, 2000), as well as shrublands (Bonanomi et al., 2008; Teste et al., 2017; Idbella et al., 2022) and grasslands (Petermann et al., 2008). However, despite such strong base, scepticism still persists especially in the community of forest ecology (Terborgh, 2020). The main doubts about the actual relevance of the JC hypothesis are caused by the variable results observed in multispecies studies reporting JC distributions for some species but Hubbell patterns for others, also named as reverse JC, and interpreted as a positive distance-dependent process due to accumulation of symbiotic microbiota (Zahra et al., 2021). Although it cannot be denied that JC patterns do exist in many species, their absence in other coexisting species raised a strong debate on the effective generality and magnitude of JC effects (Song et al., 2021). Explaining the reasons of such variability of occurrence of JC distributions would shed light on the relevance of this ecological phenomenon and on its effect on species coexistence and diversity maintenance.

In this regard, a relevant issue is whether the distance-dependent mortality factors, preventing the recruitment near the parent tree, are affected by the surrounding density of neighboring conspecific adults. This point has been mostly neglected by previous studies that focused only on the focal tree concept, overlooking the possible role of the surrounding landscape of both conspecific and heterospecific trees. Only the recently published work by Smith (2022) recognized that conspecific density may affect JC effects on species coexistence relating this to putative changes of predation levels. Theoretically, the density of conspecific adults may affect the behavior of invertebrate and vertebrate predators (Janzen, 1971), the spread of airborne and soilborne pathogens (Sapoukhina et al., 2010), as well as leaf and root litter distribution and the associated self-inhibitory factors produced during decomposition (Bonanomi et al., 2017). For example, an isolated tree accumulates litter under its canopy, thus creating a pattern associated with the concept of “island of fertility” when interpreted in terms of positive soil conditions for plant growth (Facelli and Brock, 2000), but also generating a round shaped exclusion zone around its trunk and within its own crown projection by NF. However, as the surrounding density of adult trees of the same species increases, the spatial distribution of litter progressively overlaps among individuals, generating a complex patchiness in terms of exclusion zones created by the compenetrating conspecific “litter islands”.

The aim of this work is to explore the connection between adult density, either conspecifics or heterospecifics, on the probability of occurrence of JC distributions. In detail, using an Individual-Based Modeling (IBM) approach (DeAngelis and Grimm, 2014), we simulated the formation of exclusion zones due to the build-up of NF in proximity of conspecific adult plants. The specific hypotheses of our study were:

(i) The frequency of JC distribution is high in the case of isolated trees;
(ii) The occurrence of JC distributions decreases as adult conspecific density increases due to the progressive overlap of exclusion zones;
(iii) The JC distributions are rare in the case of isolated individuals of a species when immersed in a matrix of heterospecific trees because of a dilution effect on NF conditions.

## 2 Material and methods

### 2.1 Model rationale

The model presented here was developed to investigate the role of forest stand density and species diversity on the occurrence of exclusion zones produced by localized NF. The model is developed to represent the effect of NF on seedling establishment caused by both the accumulation in the soil of the autotoxic plant self-DNA (Mazzoleni et al., 2015) and the increased attack of natural enemies such as pathogenic fungi, oomycetes and nematodes (Agrios, 2005; Van der Putten et al., 2013).

The model is based on three assumptions: i) NF is species-specific i.e., it affects only plants of the same species; ii) NF intensity is proportional to the aboveground tree biomass, and; iii) the presence of heterospecific individuals in the same area decreases the intensity of the NF. The first assumption is based on a very large number of studies demonstrating the species-specificity of this phenomenon (reviewed in Kulmatiski et al., 2008; Van der Putten et al., 2013; Cesarano et al., 2017). The second assumption is reasonable considering the autotoxicity hypothesis (Mazzoleni et al., 2007), with a release of autotoxic factors proportional to the amount of standing litter and its decay rate. Moreover, also the amount of soilborne pathogens inoculum is often proportional to the amount of plant residues left over and incorporated into the soil (Agrios, 2005; Bonanomi et al., 2007). The third assumption is based on the hypothesis of a physical dilution of conspecific autotoxic litter in mixed multispecies stands (Mazzoleni et al., 2007; Mazzoleni et al., 2010). Moreover, rare species are indirectly protected by non-host, neighboring heterospecifics, as predicted by the herd-immunity hypothesis, reducing the probability of contact with propagules of virulent plant enemies (Wills et al., 1997),

In the following sections, the model implementation and the simulation design are described.

### 2.2 Model description and simulation setup

Each simulated experiment is initialized with an area of 140 x 140 m^2^ (1.96 ha) and a predefined number of individual adult trees, randomly placed within the domain. The first individual is always placed in the centre of the domain and represents the target (focal tree) of each simulated experiment. For simplicity, every tree is assumed to have a canopy radius of 5.5 m and its biomass distribution is represented by a paraboloid function, with its maximum value at the centre of the tree crown. The latter assumption derives from the two-dimensional integration of standard 3D representations of tree crowns (Pretzsch, 2009). After this initialization step, a map of biomass distribution for each tree species is calculated as sum of the biomass occurring in every pixel. Following the assumptions defined in the model rationale, i.e., that the *NF* is proportional to the aboveground tree biomass, a map of *NF* for each species is calculated using the biomass map multiplied by a coefficient (*i_NF_*) representing the intensity of the *NF*. Seeds and seedlings are assumed to have no direct contribution to *NF* because of their negligible biomass compared to the litter produced by the adult trees.

In the case of co-occurrence of two or more tree species, in the case of biomasses of different tree species overlapping, a dilution effect was taken into account due to the presence of heterospecific litter. In detail, the *NF* for each species in each point in space is calculated as follows:


eq.1 $$NF_{i}=i_{NF}\cdot B_{i}\cdot \frac{B_{i}}{\sum_{j=1}^{n}B_{j}}$$

where *B_i_* is the biomass of the *i-th* tree species, *B_j_* are the values of biomass of other species and *n* is the number of coexisting tree species. It has to be noted that in the case of monospecific stands (*n*=1), the last equation becomes:


eq.2 $$NF_{i}=i_{NF}\cdot B_{i}$$

**Figure 2:**
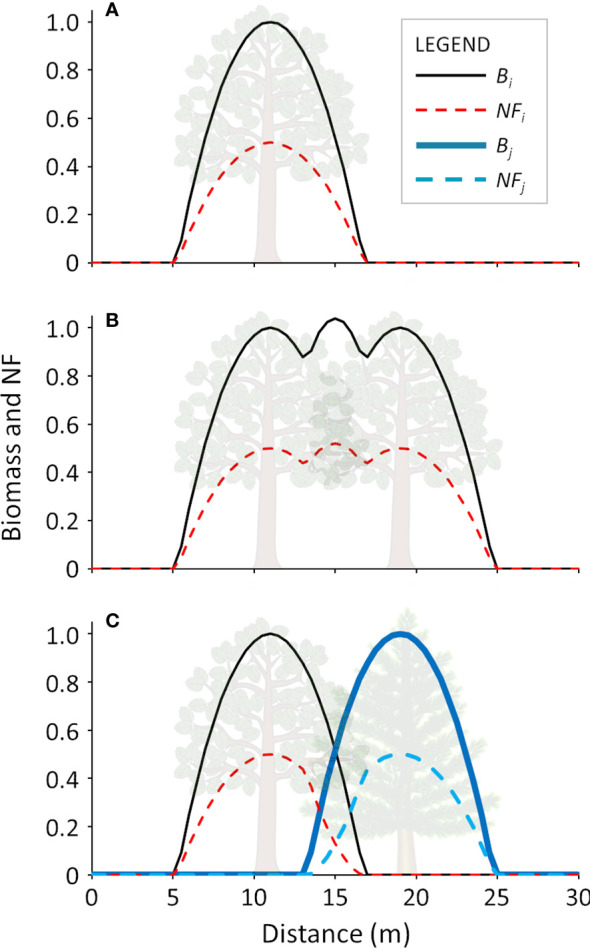
Graphical representation of calculation of tree biomass and NF in monospecific and bi-specific stands: **A**) single tree; **B**) two overlapping trees of the same species; **C**) two overlapping trees of different species. B_i_ and B_j_ represent the biomass curves, while NF_i_ and NF_j_ represent the calculated negative feedback for two generic speciIs i and j, respectively.

A visual representation of the calculation of the trees’ biomass and related *NF* is shown in **Figure 2**. Examples for an isolated tree (**Figure 2A**), overlapping trees of the same species (**Figure 2B**) and overlapping trees of different species (**Figure 2C**) are provided.

After the calculation of the biomass and *NF* maps, each individual tree is assumed to produce 1000 seeds which are distributed in the plot space using a dispersal kernel according to the Weibull distribution (Weibull, 1951). Specifically, the exponential decreasing version of the function has been chosen (Greene et al., 2004; Nathan and Casagrandi, 2004) using the following equation:


eq.3 $$f\left(x\right)=\;\frac{1}{N}e^{-Lx^{s}}$$

where *x* is the distance from the origin, *N*=1, *L*=0.1 and *s*=1. All seeds that fall outside the 140 x 140 m^2^ domain are discarded.

Then each seed of the *i-th* species has a specific probability to germinate and establish depending on the level of *NF* at the spatial location where it has fallen, calculated as follows (Vincenot et al., 2017):


eq.4 $$\;P(establishment)=\frac{\alpha }{1+\beta \cdot e^{\gamma \cdot NF_{i}}}$$

where *α=0.1*, *β=1* and *γ=5* are shape parameters and *NF_i_* is the value of *NF* for the *i-th* tree species at the specific position in space where the seed is located.

Using the procedure described above, we performed two sets of simulations, in either mono- or bi-specific stands, to study the effects of *NF* on the resulting seedlings recruitment distributions. In the case of monospecific forests, we carried out numerical experiments using six different levels of adult tree densities (1, 10, 25, 50, 100, and 200 trees in the simulated plots) factorially combined with three levels of NF intensities (*i_NF_* = 0.1, 0.5 and 1.0). Overall, 18 different scenarios of monospecific stands were produced. Moreover, an additional simulation was performed with full crown cover over the whole domain to provide a reference value for closed monospecific forest systems.

**Figure 3:**
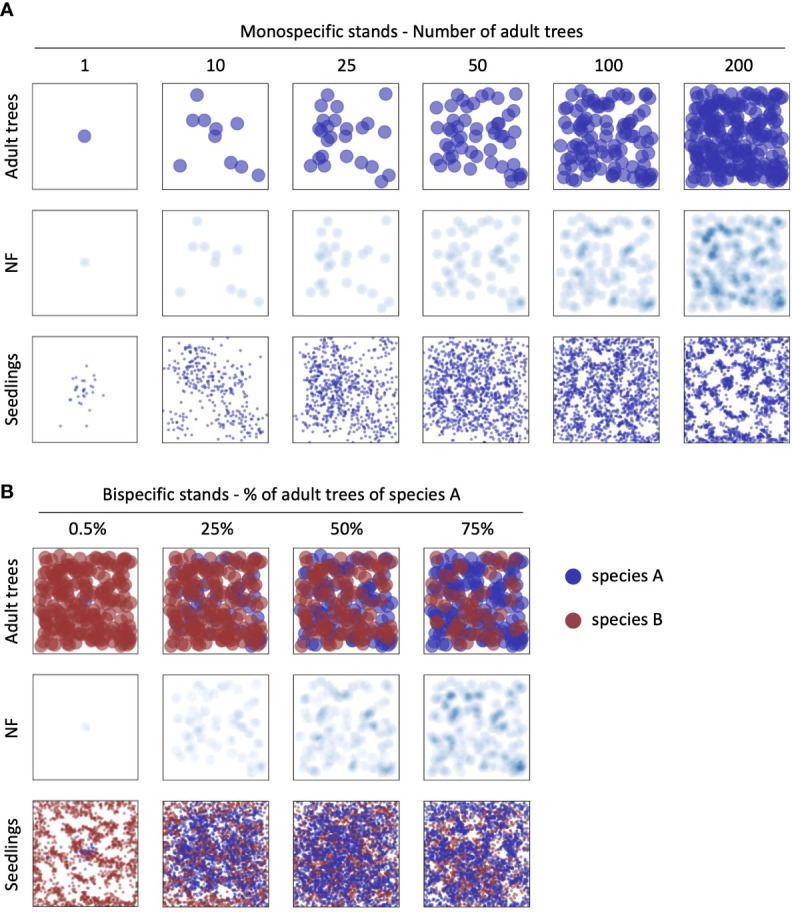
Examples of the biomass, NF, and seedlings maps in the simulated scenarios. **A**) monospecific stand simulations at increasing tree density; **B**) examples of bi-specific stand simulations in the case of 200 adult trees at different % of the two different species. All NF maps are represented using i_NF_=0.5.

In the case of bi-specific stands, we carried out the simulations with stands represented at four different adult trees densities (25, 50, 100, and 200 individuals in the simulated plots). For each density level, the concept of species replacement series (Jolliffe et al., 1984) was applied as follows: i) only one individual of species A; ii) 25% of species A and 75% of species B, iii) 50% of both species A and B, iv) 75% of species A and 25% of species B. All simulations were run with three levels of *NF* (*i_NF_* = 0.1, 0.5 and 1.0). Overall, 48 scenarios representing bispecific stands were produced. Examples of the biomass, *NF*, and seedlings distribution maps in the simulated scenarios as described above are presented in **Figure 3**.

Due to the stochastic nature of some of the modelled processes (initial tree distribution, seeds distribution and seedlings establishment), each simulated scenario was run 1000 times by a Montecarlo approach. The presented model was implemented in the Python3 programming language with standard Python libraries: math, numpy, matplotlib, random. The pseudocode of the simulation pipeline is provided in **Figure S1**.

### 2.3 Data analysis

**Figure 4:**
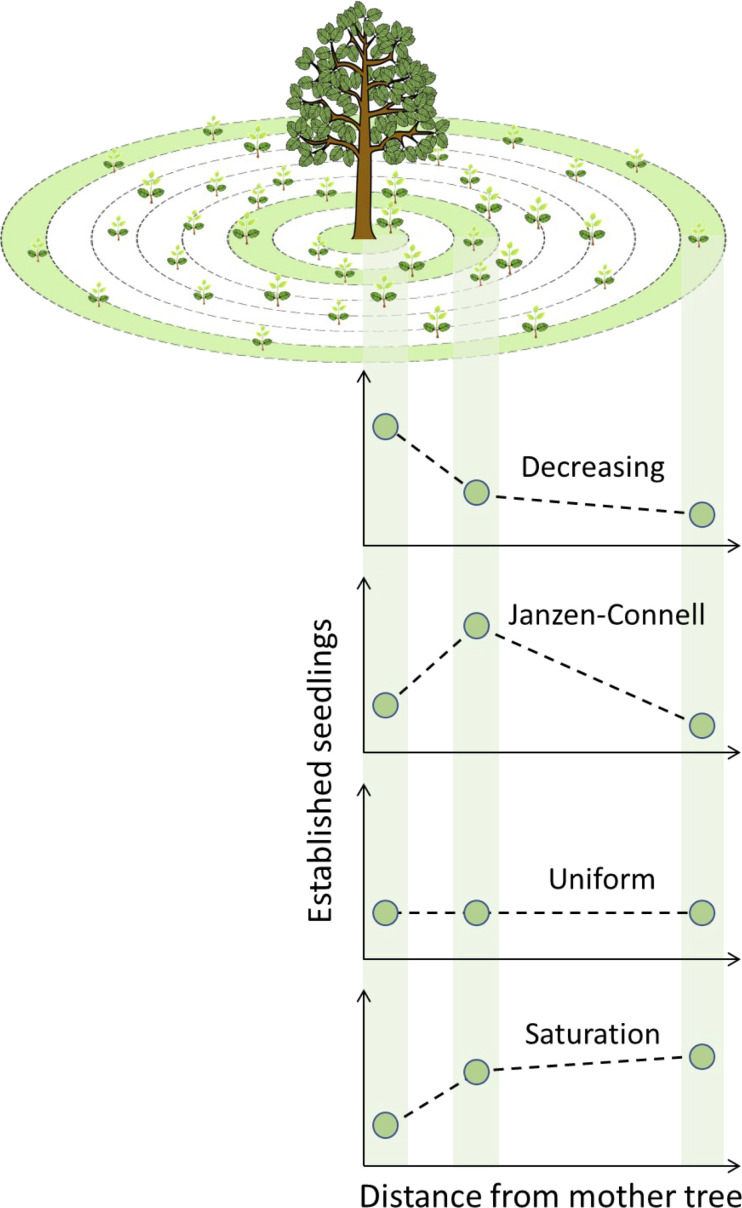
Seedling distribution calculation scheme and types of resulting recruitments patterns. Green areas indicate the three sampling areas where established seedlings were counted. After calculation of the slopes of the segments (dashed lines) between each pair of consecutive points (number of established seedlings), four recruitment patterns were defined: Decreasing, Janzen-Connell, Uniform and Saturation.

The aim of the analysis of simulation results was to quantify and classify the distribution of established seedlings of a focal tree species. Starting from the centre of the target tree located at the plot centre, the map was divided into concentric circular annuli, i.e., rings, with a 1 m width. In each annulus, the number of established seedlings after every simulation run, has been counted and normalized by the total area of the corresponding annulus. The distance-dependent recruitment patterns were classified using the average density of seedlings of three specific annuli with the following radiuses from the centre of the focal tree: 1) between 1 and 4 m, 2) between 6 and 9 m and 3) between 47 and 50 m. These three areas were selected to represent the seedling density below the crown of the mother focal tree, the area right outside its crown, and an area corresponding to the maximal seed dispersal distance. The specific domain size (140 x 140 m^2^) has been selected to avoid any significant border effect due to the lack of seeds arriving from outside the modelled domain, while keeping the model code relatively fast to execute. The specific seedling density of the three abovementioned areas are then plotted and two straight segments connecting each pair of consecutive points are constructed. We then calculate the slope (σ) of the two segments and associate each couple of possible values (σ_1_ and σ_2_) to a seedlings recruitment distribution as follows (**Figure 4**):

- σ_1_<0 and σ_2_ ≤ 0 = Decreasing distribution;
- σ_1_≥0 and σ_2_<0 = Janzen-Connell distribution;
- σ_1 _= 0 and σ_2 _= 0 = Uniform distribution;
- σ_1_>0 and σ_2_≥0 = Saturation distribution.

The case σ_1 ≤_ 0 and σ_2_>0 was never observed in any simulation. Finally, the occurrences of each recruitment pattern within each simulated scenario were counted and expressed as either relative abundance or average over the 1000 independent replicates.

## 3 Results

### 3.1 Monospecific forest stand

**Figure 5:**
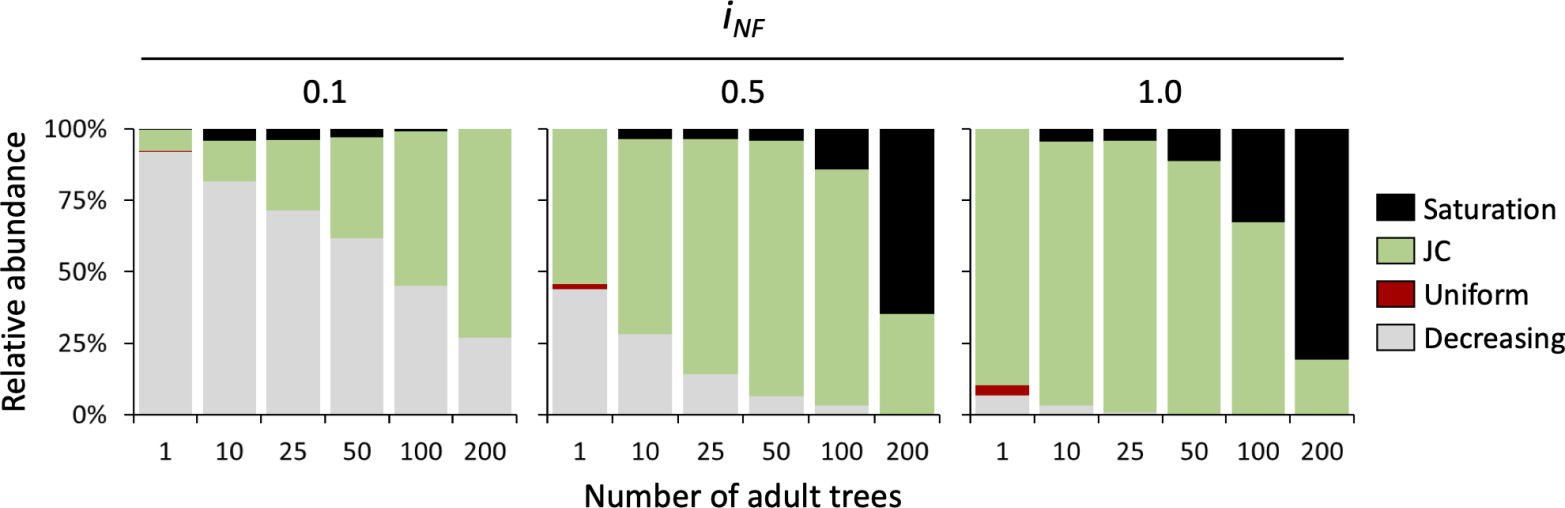
Relative distribution of seedlings recruitment pattern in monospecific stands at different levels of NF (i_NF_ = 0,1, 0.5, and 1) and density of adult tree (1, 10, 25, 50, 100 and 200 individuals per simulated plot).

In monospecific simulations with isolated trees (**Figure 5**), the JC distribution is frequent only with strong NF (*i_NF_* = 1), whereas with reduced self-inhibition decreasing distributions increase their frequency becoming dominant at low levels of NF (*i_NF_* = 0.1). Noteworthy, in monospecific communities, the occurrence of different recruitment distribution patterns is greatly affected by tree density. In detail, the probability of observing a decreasing distribution declines with increasing tree density, especially at medium and strong NF levels (*i_NF_* = 0.5 and 1). We observed a smooth and gradual reduction in the occurrence of the decreasing distribution at low levels of NF (*i_NF_* = 0.1), which was replaced by the JC recruitment pattern. At high NF intensity (*i_NF_* = 1), we observed a similar trend, but with the replacement of the decreasing distribution by a saturation distribution proportional to tree density. At high tree density (200 trees), representing a forest with continuous and dense cover (see **Figure 3A**, last column), the occurrence of the JC distribution decreases with increasing NF, reaching only 19.4% of cases at the highest NF intensity. A uniform distribution was found in very few cases (less than 5% of the simulations) and only in the condition of isolated trees with medium and strong NF levels (**Figure 5**).

When all the 1000 permutations were averaged (**Figure S2**), the results confirmed what was observed in terms of frequencies of establishment patterns. Considering the case of low NF intensity (**Figure S2** upper panel) the average distributions of seedlings assume the shape of a decreasing pattern even when more than 50% of the cases were classified as JC at high tree density (100 and 200 trees in **Figure 5**). For higher values of NF intensity (*i_NF_* = 0.5 and 1), almost all average curves are classified as JC distributions with the only exception of the highest density (200 trees) where the resulting patterns are saturation (i.e., increasing number of established seedlings with distance from the mother tree). In terms of absolute numbers of established seedlings, a clear pattern emerges, i.e., the establishment decreases strongly with the increase of the NF intensity (**Figure S2**). In all simulations, even at the highest tree density (200 trees), there is presence of bare soil among tree crowns in the plot (**Figure 3A**). Differently, in the case of simulations performed imposing a full coverage of the plot, the constant accumulation of NF all over the domain, produces the disappearance of any spatial pattern of seedlings establishment and, in particular, the absence of observable JC distribution (red lines in **Figure S2**).

### 3.2 Mixed forest stand

**Figure 6:**
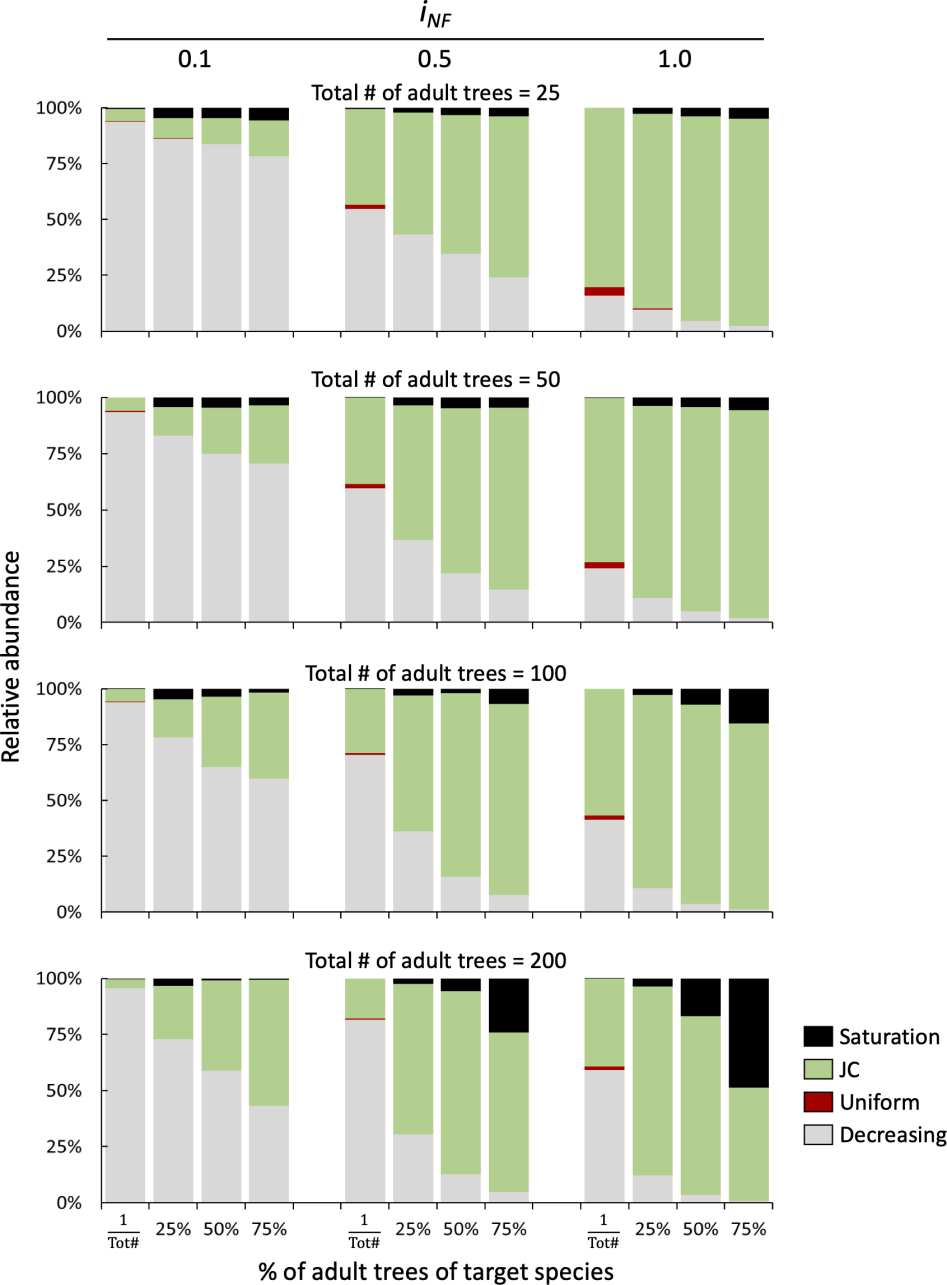
Relative distribution of seedlings recruitment patterns in bi-specific stands at different levels of NF (i_NF_ = 0,1, 0.5, and 1) and density of adult tree (25, 50, 100 and 200). The x-axis shows the species replacement series starting with only one individual (first column) followed by 25%, 50% and 75% of individuals of the target species.

In the case of mixed two-species stands, rare species immersed in a matrix of heterospecifics rarely shows JC distributions with decreasing recruitment pattern predominating, especially at low and medium NF intensity (*i_NF_* =0.1 and 0.5), (first column of each bar plot in **Figure 6**). In the case of a co-dominated community with a forest stand composed by 50% species A and 50% species B, both tree density and NF affect the probability of observing a JC distribution. At low tree density (representing a Savannah ecosystem), with 25 and 50 total trees, the low NF simulation (*i_NF_* =0.1) showed that the decreasing distribution is the most frequent and only few cases of occurrence of the saturation pattern. However, as NF intensity increases (*i_NF_* =0.5 and 1.0), the JC distribution replaces the decreasing distribution, becoming the most frequent with over 80% of the cases. In co-dominated stands with high cover (either 100 or 200 trees per plot), JC and decreasing distributions are almost equally likely to occur at low NF level (*i_NF_* =0.1). Instead, on one hand, when NF is medium or high (*i_NF_* =0.5 and 1.0), JC distribution is observed in more than 80% of simulations with few cases with either decreasing or uniform recruitment patterns. On the other hand, in stands with high density (200 trees) and dominated by the target species (75% of cover), the probability of observing JC recruitment is more than 50% regardless of NF intensity, reaching the highest value (71.1%) at medium NF intensity level (**Figure 6**).

The presented results are confirmed when the average of the 1000 permutations are considered (**Figure S3**). The most abundant establishment pattern is a decreasing distribution at low NF intensity (*i_NF_* =0.1), while the JC pattern gradually appears with increasing NF levels (*i_NF_* =0.5 and 1.0). Also in this case, as was observed for the monospecific stands, the absolute density of seedlings decreases strongly with increasing NF intensity (**Figure S3**).

## 4 Discussion

Assuming that NF is species-specific, localized under an individual tree, and with limited horizontal diffusion, our simulations show that tree density is critical to understand the observed variability of tree recruitment patterns. Our model highlights the complex interconnection between NF intensity, stand density, and recruitment patterns explaining where and why the JC distribution occurs, and clarifying the relevance of this ecological phenomenon in different plant community frameworks.

Our initial hypothesis that JC distribution is very common in the case of an isolated tree was partially supported by the model simulations. Indeed, we found that JC distribution was very frequent for isolated trees when NF was strong and capable to form an exclusion zone under the parent tree. However, with decreasing NF intensity, both JC and decreasing patterns co-occurred and were recorded with similar frequencies. A prevalence of the decreasing pattern was also observed at very low NF, because under such conditions the inhibitory effect due to NF was unable to overcome the clustering effect of the seed dispersal kernel, with resulting concentrated recruitment under the parent trees. JC distribution in isolated individuals has been previously reported for both shrubs and trees, but in far less cases compared to tropical and temperate forests (reviewed in Bonanomi et al., 2010; Comita et al., 2014; Song et al., 2021). A notable example is the study of Clark & Clark (1981), reporting a clear distance-dependent recruitment limitation for isolated trees of *Bursera graveolens* in arid ecosystems with discontinuous vegetation. Moreover, a recruitment distribution consistent with the JC model has been reported for woody plants belonging to Fabaceae, a plant family forming fertility islands under individual canopies, associated to the accumulation of organic matter, nitrogen and phosphorus (Facelli & Brock, 2000). Under this scenario, in order to observe a distance-dependent inhibition, the generating factors of NF, attributed either to soilborne pathogens or soil autotoxicity, must overwhelm the positive effects of both nutrients and beneficial microbes of the fertility islands under the canopy of woody plants. In this context, it is well established that plants belonging to Fabaceae suffer greatly from NF in both agroecosystems and natural plant communities (Cesarano et al., 2017). Accordingly, several studies reported the presence of intense NF and JC recruitment distributions in woody perennial plants, including *Medicago marina* in Mediterranean sand dunes (Bonanomi et al., 2008), *Medicago sativa* in US old field (Jennings & Nelson, 2002), *Genista aetnensis* over volcanic lavas (Stinca et al., 2015), and several *Acacia* species in South Africa (Ben-Shahar, 1991).

Regarding the second hypothesis, i.e., the decreasing occurrence probability of JC distribution with high density of conspecifics, our model demonstrated a complex scenario dependent on the intensity of the NF. Indeed, when the NF intensity is low, the JC frequency increases linearly with the density of adult conspecifics. However, if NF is strong, the peaks in JC frequency are still observed at intermediate stand densities while suddenly decrease in stands with a continuous conspecific cover of 200 trees in the plot. So, counterintuitively, our model shows that a plant suffering from strong NF in monospecific stands can rarely exhibit a recruitment pattern fitting the JC model. This seemingly paradoxical result is due to the progressive expansion of the exclusion zone surrounding all trees in the forest stand. In other words, as individual trees become more clustered and denser, their exclusion zones progressively overlap, leaving no safe place for an effective recruitment in the stand. This is consistent with the lack of JC evidence in many monospecific stands in temperate and boreal forests, including *Fagus sylvatica* in Mediterranean forests (Rita et al., 2021), as well as monodominant tropical forests (Hart et al., 1989; Richards, 1996). In general terms, our model demonstrates the association between strong NF and lack of distance-dependent inhibition in dense, monospecific stands. This result reconciles NF with forest composition and should reduce the scepticism of many forest ecologists towards the JC model (Terborgh, 2020).

Our third hypothesis supposed that the JC distribution should not be frequent in the case of rare species immersed in a matrix of heterospecific adults. This was largely confirmed by our numerical simulations demonstrating that a species with only 25% stand cover showed lower frequency of JC distribution compared to stands in which the species occurrence was at 50% and 75% cover. This effect was observed in both low and strong NF conditions, and reflected the fact that the abundant presence of heterospecific adult neighbors provides a suitable place for recruitment overlapping with the exclusion zones by conspecifics and thus reducing the NF effect. Rare species are indirectly protected by non-host, neighboring heterospecifics, as predicted by the herd-immunity hypothesis, which reduces the probability of contact with propagules of the virulent plant enemies (Wills et al., 1997). In the case of the autotoxicity theory, the presence of leaf litter and root debris from heterospecifics likely results in the dilution of conspecific plant residues, thus providing soil patches free of NF even near conspecific mother plants.

With respect to forest dynamics, our model simulations are consistent with robust field data on alternative species replacement reported in temperate and boreal forests around the globe, in stands co-dominated by two tree species (Fox, 1977). Notable examples include *Fagus grandifolia* with *Acer saccharum*, *Picea rubens* with *Abies fraseri*, *Fagus grandifolia* with *Tsuga canadensis*, and *Picea engelmannii* with *Abies lasiocarpa* (Whittaker & Levin, 1977; Woods, 1979; Runkle, 1981; Waters & Savill, 1992). In all these studies, the recruitment of tree species was found to be significantly more abundant and healthy under heterospecific adults. In this context, on one hand our model shows that localized NF is able to explain species replacement in forest ecosystems. On the other hand, the observation of decreasing JC recruitment patterns in co-dominated mixed forests reflects a reduced NF effect related to departure from monospecificity.

Future model simulations can focus on multispecies systems to test the effect of adult density on distance-dependent inhibition also in forest stands with high tree diversity and also assess the fate of rare species having different levels of sensitivity to NF compared to the most abundant plant in the community (Van der Putten et al., 2013). Spatial comparison of numerical simulations with real data obtained from long-term forest censuses for tropical forests such as Barro Colorado (Condit, 1998), other tropical forests (Lewis et al., 2004), and temperate forests (Král et al., 2018) could be particularly useful to this goal.

From a modelling point of view, future work could address the following points: (i) explicit representation of the germination and establishment processes separately to help disentangle the effect of different causal mechanisms due to either chemical autotoxicity or action of soil-borne pathogens. Specifically, the effect of chemical autotoxicity is reported to affect both germination and early seedlings’ growth, whereas soil-borne pathogens mostly affect the establishment phase. (ii) In order to provide a more general description of plant-soil interactions, the inclusion of facilitation by heterospecific biomass can be also explicitly considered to evaluate its relevance in the emergence of seedlings’ JC patterns. (iii) To test the effect species-specific characteristics like crown shape and seed dispersal strategies on the emergence of seedlings’ establishment patterns, different formulations of the biomass distribution and seed dispersal kernels could be implemented. (iv) Moreover, future studies could investigate the impact of the priority effect by simulating different colonization timings, as occurs in ecological succession, and the consequent effect on the recruitment of conspecifics.

Finally, a challenge for future studies will be a spatially explicit definition of the exclusion zone in forests with different tree density and diversity. New-generation sequencing techniques may enable the production of fine-scale metagenomic maps coupled with an assessment of the conspecific extracellular DNA accumulated in the soil where the NF effects are observed. Such in-depth investigations on spatial information associated to tree recruitment distribution will be relevant to support the discussion on the putative mechanisms of the JC effect and to disentangle between the hypotheses of soilborne pathogens and self-DNA inhibitory effects, thus providing a better understanding of the spatial and temporal patterns of this important phenomenon.

## Data availability statement

The data that support the findings of this study are available from the corresponding author upon reasonable request.

## Author contributions

GB conceived the work and performed data analysis. AB implemented the model and performed numerical simulations. FC designed and prepared the figures and designed the numerical simulations. SM conceived the work and designed the model and data analysis. FG designed the model, the numerical simulations and data analysis and team coordination. All authors contributed to the article and approved the submitted version.

## Supplementary material

Supplementary Figure 1 Pseudocode describing the algorithm used in our model. It consists of two main parts: calculation of Seeds/Seedlings maps (Algoritm_1), and analysis of these distributions and classification (Algoritm_2).

Supplementary Figure 2 Normalized number of seedlings along a transect from the mother tree in monospecific stands at different levels of NF (i_NF_ = 0,1, 0.5, and 1) and density of adult tree (1, 10, 25, 50, 100 and 200 individuals per simulated plot). Light grey areas represent the extent of the mother tree’s crown. Red lines represent establishment levels with full crown coverage. Normalization performed dividing the number of established seedlings by the maximum number of established seedlings recorded among all simulations.

Supplementary Figure 3 Normalized number of seedlings along a transect from the mother tree in bi-specific stands at different levels of NF (i_NF_ = 0,1, 0.5, and 1) and density of adult tree (25, 50, 100 and 200). The different curves within each plot represent the species replacement series starting with only one individual (dashed grey line) followed by 25% (solid grey line), 50% (dashed black line) and 75% (solid black line) of individuals of the target species. Light grey areas represent the extent of the mother tree’s crown. Normalization performed dividing the number of established seedlings by the maximum number of established seedlings recorded among all simulations.
