# Supplementary material for: Adult conspecific density affects Janzen-Connell patterns by modulating the recruitment exclusion zones
Source: Front Plant Sci. 2023 Jun 27;14:1079975. doi: 10.3389/fpls.2023.1079975 (PMC10333542; doi:10.3389/fpls.2023.1079975)
Supplement: Supplementary Figure 1 — Pseudocode describing the algorithm used in our model. It consists of two main parts: calculation of Seeds/Seedlings maps (Algoritm_1), and analysis of these distributions and classification (Algoritm_2). [file Image_1.pdf]

Algorithm_1: Seeds and Seedlings maps

```
% Input Algorithm_1 setup
Define Number of species (1 or 2)
Define Number of trees
Define NF level
Define number of iterations
% Initialization stage
   Target Tree in the center
For each iterations
   For each tree
      Random Tree positions
   Endfor
   % NF map
   Calculate NF matrix
   % seed and seedling map
   For each tree
      Produce 1000 Seeds
      Seed dispersal
      Seed establishment (Seedling)
   Endfor
Endfor
```

Algorithm_2: Seeds and Seedlings distribution and classification

```
% Input Algorithm_2 setup
Define tori
Define tori areas
Define 4 groups: Janzen-Connel; Uniform; Hubbel; Saturation
% Calculate Seeds and Seedlings distributions
For each of 1000 iterations
   For each tori
      Count number of seeds
      Count number od seedlings
   Endfor
Normalization seeds and seedlings distribution
Classification each distribution into 4 groups
Endfor
```
