# Supplementary figures and images for: Adult conspecific density affects Janzen-Connell patterns by modulating the recruitment exclusion zones
Source: Front Plant Sci. 2023 Jun 27;14:1079975. doi: 10.3389/fpls.2023.1079975 (PMC10333542; doi:10.3389/fpls.2023.1079975)

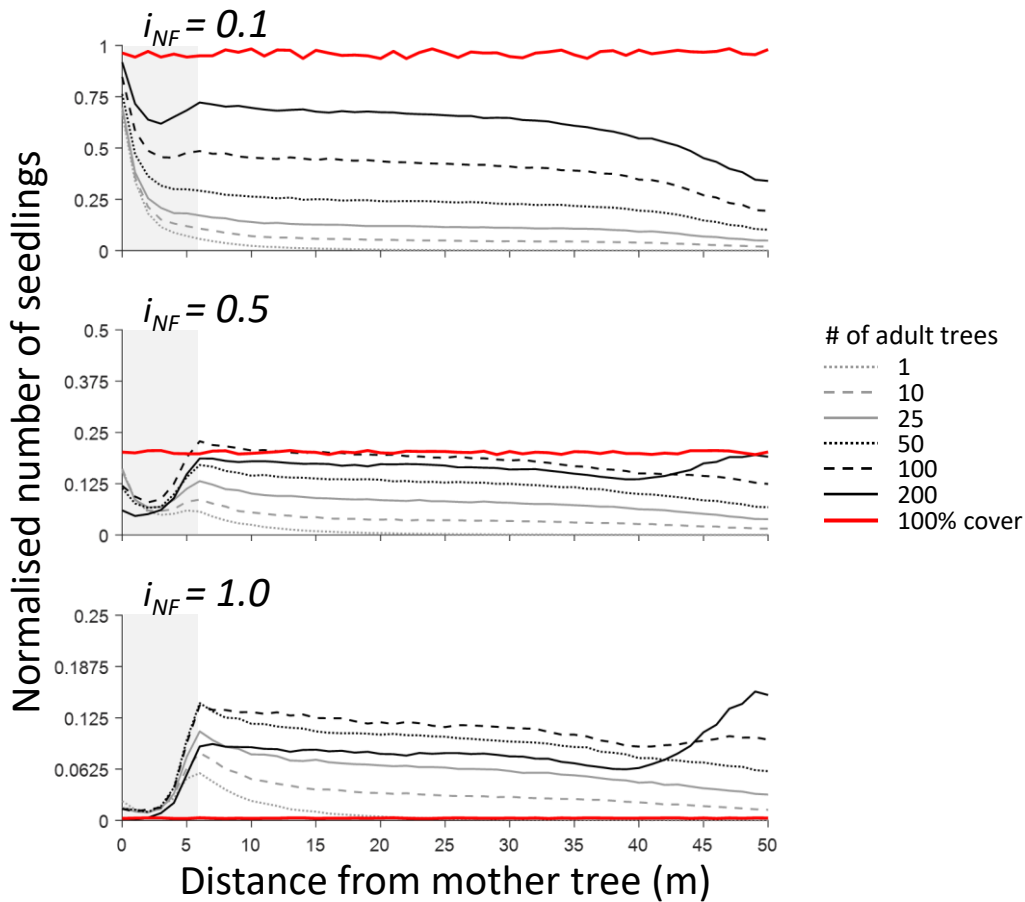

Supplement: Supplementary Figure 2 — Normalized number of seedlings along a transect from the mother tree in monospecific stands at different levels of NF (iNF = 0,1, 0.5, and 1) and density of adult tree (1, 10, 25, 50, 100 and 200 individuals per simulated plot). Light grey areas represent the extent of the mother tree’s crown. Red lines represent establishment levels with full crown coverage. Normalization performed dividing the number of established seedlings by the maximum number of established seedlings recorded among all simulations. [file Image_2.pdf]
