# Supplementary material for: Adult conspecific density affects Janzen-Connell patterns by modulating the recruitment exclusion zones
Source: Front Plant Sci. 2023 Jun 27;14:1079975. doi: 10.3389/fpls.2023.1079975 (PMC10333542; doi:10.3389/fpls.2023.1079975)
Supplement: Supplementary Figure 3 — Normalized number of seedlings along a transect from the mother tree in bi-specific stands at different levels of NF (iNF = 0,1, 0.5, and 1) and density of adult tree (25, 50, 100 and 200). The different curves within each plot represent the species replacement series starting with only one individual (dashed grey line) followed by 25% (solid grey line), 50% (dashed black line) and 75% (solid black line) of individuals of the target species. Light grey areas represent the extent of the mother tree’s crown. Normalization performed dividing the number of established seedlings by the maximum number of established seedlings recorded among all simulations. [file Image_3.pdf]

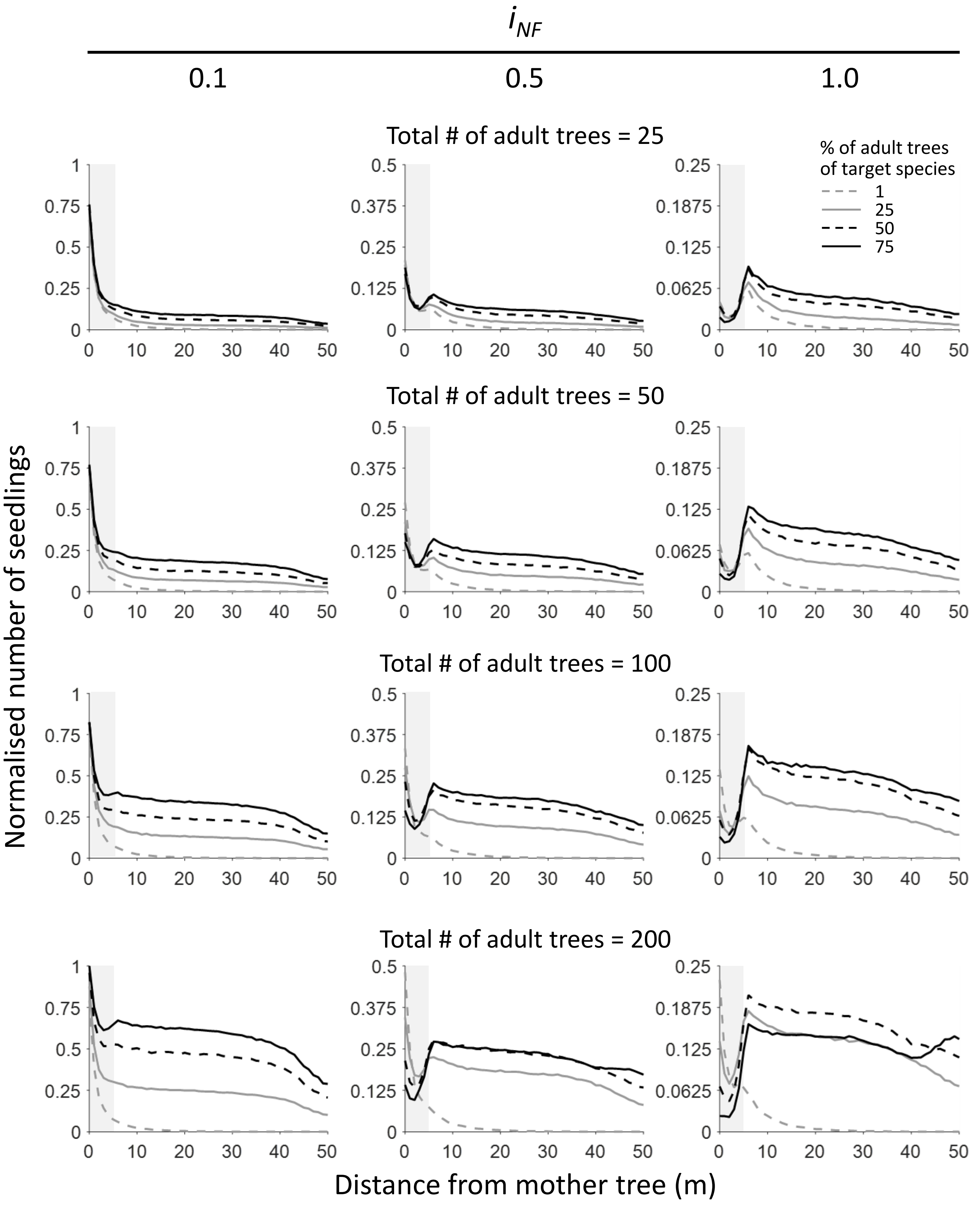

i_{NF}
0.1
0.5
1.0
Total # of adult trees = 25
% of adult trees of target species
1
25
50
75
Total # of adult trees = 50
Total # of adult trees = 100
Total # of adult trees = 200
Normalised number of seedlings
Distance from mother tree (m)
